# Impact of Dialysis Method on Colon Postpolypectomy Bleeding in Patients with End-stage Renal Disease

**DOI:** 10.5152/tjg.2024.23640

**Published:** 2024-08-01

**Authors:** Hsueh-Chien Chiang, Chien-Ming Chiang, Xi-Zhang Lin, Po-Jun Chen

**Affiliations:** 1 Department of Internal Medicine, National Cheng Kung University Hospital, College of Medicine, National Cheng Kung University, Tainan, Taiwan; 2 Institute of Clinical Medicine College of Medicine, National Cheng Kung University, Tainan, Taiwan

Dear Editor,

Postpolypectomy bleeding is the most common complication of colonoscopic polypectomy, with a risk of approximately 1%-3%.^1^ There are 2 types of postpolypectomy bleeding—immediate postpolypectomy bleeding, which is intraprocedural bleeding that can be managed during colonoscopy, and delayed postpolypectomy bleeding (DPB), which is bleeding that occurs within 30 days after the endoscopy.^2^

Age, gender, polyp location, polyp morphology, antithrombotic medication use, and polypectomy method are known risk factors for DPB.^3^ Additionally, patients who have end-stage renal disease (ESRD) are at a higher risk of postpolypectomy bleeding.^4^ Uremic platelet dysfunction, renal anemia, poor antiplatelet agent clearance, and anticoagulation during dialysis increase the risk of bleeding in ESRD patients.^5^ Peritoneal dialysis (PD) and hemodialysis (HD) are the most common dialysis methods worldwide. However, which dialysis method has the higher risk of DPB is unknown. This cohort study will compare the incident rate of DPB between patients undergoing PD and HD.

This is a retrospective cohort study conducted at a single tertiary medical center. The study protocol was approved by the Institutional Review Board of National Cheng Kung University Hospital (approval number: B-ER-112-230, date: August 4, 2023). Written informed consent was obtained from the patients who agreed to take part in the study. Patients with ESRD who underwent colonoscopic polypectomy during the period from January 2014 to January 2023 at our hospital were included. Patients who received antiplatelet agents or anticoagulants without adequate discontinuation, patients who lost follow-up within 30 days, and patients with a maximal colon polyp size less than 0.5 cm were excluded. The patients’ baseline characteristics, comorbidities, dialysis methods, colonoscopy procedures, serum platelet counts, and prothrombin time international normalized ratio (PT-INR) were obtained. All endoscopists had at least 2 years of colonoscopy experience. All patients were regularly followed up for 30 days.

The primary outcome was DPB from a polypectomy wound within 30 days. Delayed postpolypectomy bleeding was defined as continuous hematochezia followed by a confirmation colonoscopic examination and subsequent endoscopic hemostasis.^2^

We applied Student’s *t*-test, Pearson’s *χ* ^2^ test, and Fisher’s exact test to evaluate data regarding the patient’s characteristics and outcomes. Logistic regression analysis was applied to compare the differences in DPB between the 2 groups. A propensity score was calculated from logistic regression with the group as the dependent variable and age group, gender, BMI, antiplatelet/anticoagulant agent use, cirrhosis, hemogram, platelet, and prothrombin time as independent variables. We applied the stabilized inverse probability of treatment weighting (IPTW) method according to the generalized multiple propensity scores. For the stabilized IPTW method, a pseudo/population was created by weighting the inverse probability of a patient based on the propensity score. The calculated propensity scores were weighted using the “ratio of patients on HD to all patients/propensity score” in the HD group and the “ratio of patients receiving PD to all patients/1 propensity score” in patients treated with PD as the weighting coefficient on stability. All statistical analyses were conducted using the Statistical Package for the Social Sciences version 25.0, (IBM Corp., Armonk, NY, USA) or SAS (version 9.4; SAS Institute, Cary, NC, USA). All tests with *P* ≤ .05 are considered statistically significant.

A total of 680 patients with ESRD who underwent polypectomy during the period from January 1, 2014, to January 31, 2023, were enrolled in the study. Among these patients, 12 were lost to follow-up within 30 days, and 111 had colon polyps smaller than 5 mm and were therefore excluded. Among the remaining 557 patients, 474 underwent HD and 83 underwent PD during the colonoscopy (Figure 1). Table 1 demonstrates the patients’ baseline characteristics with HD and PD. Differences were observed in age, platelet counts, use of antiplatelet agents, and polypectomy methods between the 2 groups (Table 1).

According to the results, 127 patients (22.8%) experienced immediate postpolypectomy bleeding, and 27 patients (4.8%) experienced DPB within 30 days. Nevertheless, the rate of immediate postpolypectomy bleeding was similar between the 2 groups (22.7% vs. 22.9%, *P* = .983). However, the rate of DPB was significantly higher in the HD group than in the PD group (5.6% vs. 0%, *P* = .023, Table 1). The time to DPB was shorter in the HD group (*P* = .027, Figure 2).

To minimize the influence of patient characteristics, we applied the stabilized IPTW method based on the propensity scores. After applying the stabilized IPTW method, the rate of DPB was still significantly higher in the HD group than in the PD group (5.8% vs. 0%, *P* = .036). These results indicated that, compared with PD, HD was associated with a higher risk of DPB.

End-stage renal disease is a well-known risk factor for postpolypectomy bleeding and perforation. In our study, we discovered that compared with PD, ESRD patients on HD had a higher risk of DPB within 30 days.

Several issues can explain the higher bleeding risk of DPB in HD patients. Low-molecular weight heparin should be applied during HD sessions to prevent clotting in the extracorporeal system, which will increase the circulating heparin levels of the HD patient.^6^ Additionally, exposure of the blood to the dialysis tubes and dialyzer membrane during HD can induce complement activation-induced thrombocytopenia and platelet dysfunction.^7^ Moreover, patients with HD have higher oxidative stress by uremia than PD patients and the excessive reactive oxygen species can cause mucosal damage.^7^ Last but not least, patients with PD are younger with fewer comorbidities and less drug use. Before the polypectomy wound heals, these factors can cause the wound to rebleed, especially in a larger wound with visible vessels.

Several limitations remained in this study. Being a retrospective cohort study, the choice of polypectomy technique and the choice of utilizing prophylactic clipping were based on the operator’s preference and experience. Additionally, PD is associated with better physical ability, cognitive function, and family support. Although shared decision-making helps ESRD patients decide on their dialysis method, nephrologists suggest against PD for patients’ lack of self-care. We also found the patient number on antiplatelet agents was imbalanced between the 2 groups, which may be due to the prevention effect on vascular access failure by antiplatelets. However, all enrolled patients discontinued their antiplatelet agents for 5-7 days before the colonoscopy. Additionally, after stabilized IPTW, all baseline characteristics were balanced. Moreover, the patient numbers between the PD and HD group were with a great disparity. Although we applied propensity score-based IPTW to reduce the selection bias to a minimum and to adjust patient baseline characteristics, there may be unknown confounding factors.

In conclusion, this is the first study to indicate that HD is associated with a higher risk of DPB compared to PD in ESRD patients. Taiwan has a high incidence rate of ESRD patients, so this finding can serve as a reference for endoscopists with limited experience in managing patients with ESRD. Nevertheless, our study results should be confirmed by more robust prospective cohort studies.

## Figures and Tables

**Figure 1.**
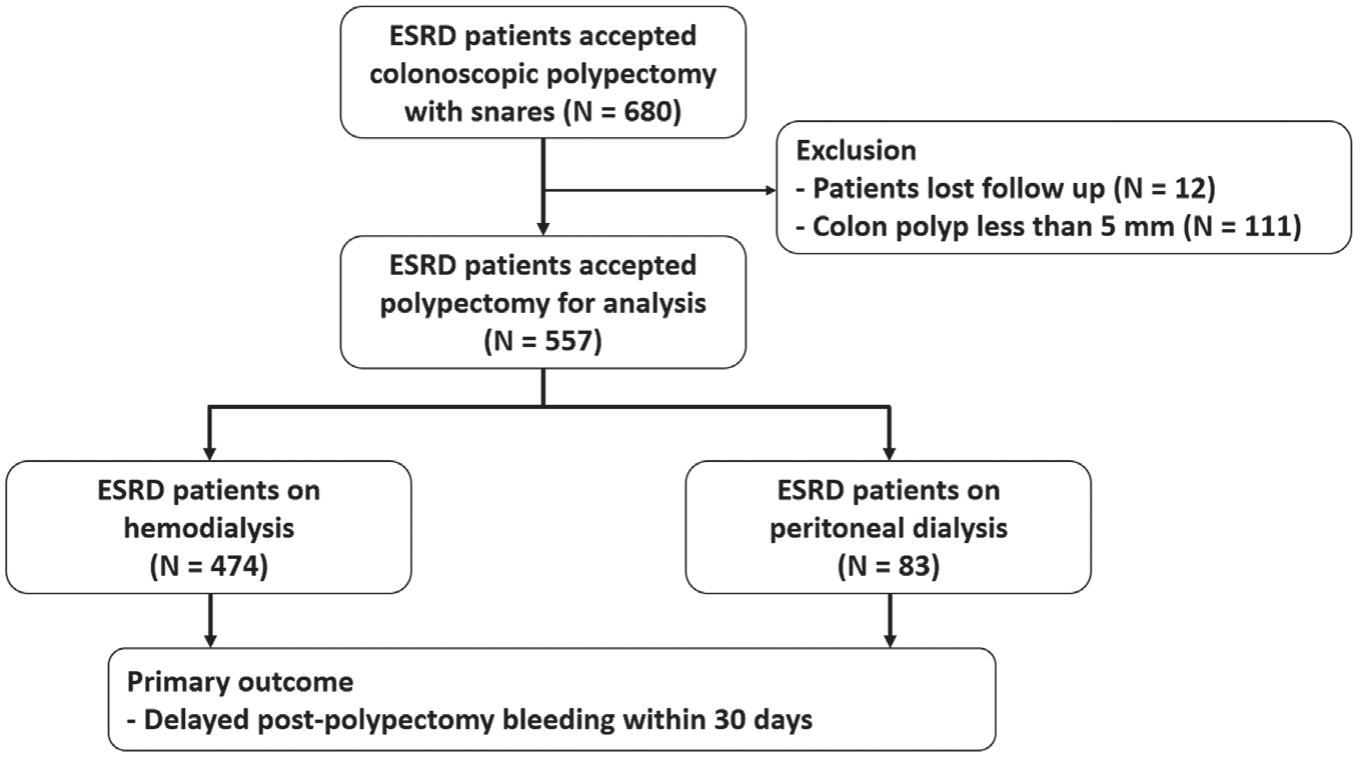
The flowchart of patient enrollment.

**Figure 2.**
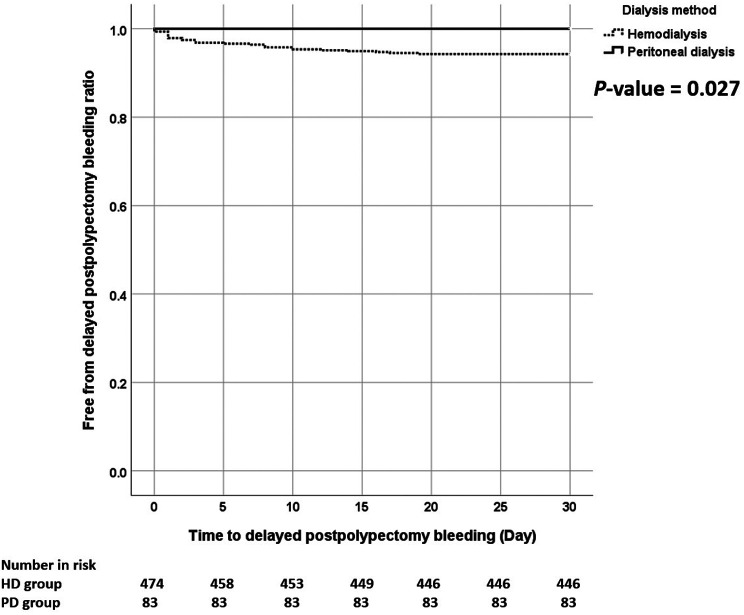
The Kaplan–Meier curve compared the period to delayed postpolypectomy bleeding between patients with HD and PD.

**Table 1.** Patient Characteristics of the 2 Types of Dialysis Methods

N = 557	Before IPTW			After IPTW^α^		
	HD (n = 474)	PD (n = 83)	*P*	HD	PD	*P*
	n (%)	n (%)		%	%	
Age (years, mean ±SD)	66.2 ± 9.5	58.0 ± 11.0	.001	64.9 ± 10.4	64.4 ± 9.2	.698
Gender			.694			.657
Male	294 (62.0)	49 (59.0)		61.5	65.1	
Female	180 (38.0)	34 (41.0)		38.5	34.9	
BMI (mean ±SD)	23.4 ± 3.7	24.3 ± 3.9	.051	23.6 ± 3.8	23.9 ± 3.5	.568
Hemoglobin (g/dL, mean ±SD)^*^	10.2 ± 1.5	10.2 ± 1.8	1.000	10.2 ± 1.6	10.0 ± 1.8	.240
Platelet (10^3^/μL, mean ±SD)^†^	185 ± 66	229 ± 93	.001	192 ± 70	195 ± 75	.700
PT-INR (mean ±SD)^‡^	1.0 ± 0.4	1.0 ± 0.1	.430	1.1 ± 0.4	1.0 ± 0.1	.385
Use of aspirin	95 (20.0)	8 (9.6)	.036	19.8	11.7	.146
Use of clopidogrel	80 (16.9)	4 (4.8)	.008	16.8	9.6	.172
Use of warfarin	7 (1.5)	0 (0.0)	.601	1.5	0.0	.602
Cirrhosis	37 (7.8)	2 (2.4)	.123	7.0	2.6	.205
Polyp size (mm, mean ±SD)	10.7 ± 4.9	10.4 ± 6.4	.653	10.8 ± 5.1	9.6 ± 5.1	.079
Polyp size >10 mm	167 (35.2)	25 (30.1)	.436	35.3	29.1	.384
Polyp type			.540			.391
Non-pedunculated	420 (88.6)	71 (85.5)		88.2	92.5	
Pedunculated	54 (11.4)	12 (14.5)		11.8	7.5	
Polypectomy method			.034			.004
Cold snare	162 (34.2)	39 (47.0)		34.0	52.6	
Hot snare	312 (65.8)	44 (53.0)		66.0	47.4	
Immediate bleeding (%)	108 (22.8)	19 (22.9)	1.000	23.1	30.8	.213
Prophylactic clipping (%)	317 (66.9)	49 (59.0)	.207	67.6	60.1	.269
Delayed bleeding (%)	27 (5.7)	0 (0.0)	.023	5.8	0.0	.036

^α^Stabilized IPTW-ATE.

^*^Seventeen patients had missing data on baseline hemoglobin.

^†^One patient had missing data on baseline platelets.

^‡^Seven patients had missing data on baseline PT-INR.
